# A wide range of pheromone-stimulated sexual and reproductive behaviors in female mice depend on G protein Gαo

**DOI:** 10.1186/1741-7007-12-31

**Published:** 2014-05-02

**Authors:** Livio Oboti, Anabel Pérez-Gómez, Matthieu Keller, Eric Jacobi, Lutz Birnbaumer, Trese Leinders-Zufall, Frank Zufall, Pablo Chamero

**Affiliations:** 1 Department of Physiology, University of Saarland School of Medicine, 66421 Homburg, Germany; 2 Laboratoire de Physiologie de la Reproduction & des Comportments, UMR 7247 INRA-CNRS-Université de Tours, F-37380 Nouzilly, France; 3 Laboratory of Neurobiology, National Institute of Environmental Health Sciences, Research Triangle Park, NC 27709, USA; 4 Present address: Children’s National Health System - Center for Neuroscience Research (CNR), Washington, DC 20310, USA; 5 Present address: Deutsches Zentrum für Neurodegenerative Erkrankungen (DZNE), 69120 Heidelberg, Germany

**Keywords:** Bruce effect, Estrus induction, Gαo signaling, Lordosis, Mate recognition, Puberty acceleration, Reproduction

## Abstract

**Background:**

Optimal reproductive fitness is essential for the biological success and survival of species. The vomeronasal organ is strongly implicated in the display of sexual and reproductive behaviors in female mice, yet the roles that apical and basal vomeronasal neuron populations play in controlling these gender-specific behaviors remain largely unclear.

**Results:**

To dissect the neural pathways underlying these functions, we genetically inactivated the basal vomeronasal organ layer using conditional, cell-specific ablation of the G protein Gαo. Female mice mutant for Gαo show severe alterations in sexual and reproductive behaviors, timing of puberty onset, and estrous cycle. These mutant mice are insensitive to reproductive facilitation stimulated by male pheromones that accelerate puberty and induce ovulation. Gαo-mutant females exhibit a striking reduction in sexual receptivity or lordosis behavior to males, but gender discrimination seems to be intact. These mice also show a loss in male scent preference, which requires a learned association for volatile olfactory signals with other nonvolatile ownership signals that are contained in the high molecular weight fraction of male urine. Thus, Gαo impacts on both instinctive and learned social responses to pheromones.

**Conclusions:**

These results highlight that sensory neurons of the Gαo-expressing vomeronasal subsystem, together with the receptors they express and the molecular cues they detect, control a wide range of fundamental mating and reproductive behaviors in female mice.

## Background

Chemical signals detected by the olfactory system can alter the reproductive state and sexual behavior of female mice. Pheromones emitted by male conspecifics elicit rapid responses in females’ behavior - such as lordosis [1,2], or sexual attraction to males [3,4] - that facilitate sexual contact. Such cues also produce long-lasting effects on endocrine physiology, including estrus-inducing events that improve the likelihood of fecundation [5,6]. Within the different olfactory subsystems [7], the vomeronasal organ (VNO) appears to be critical for the display of female reproductive behaviors. Surgical lesions of the VNO eliminate important olfactory-dependent neuroendocrine functions in female mice, including mate recognition and pregnancy block, facilitation of lordosis, puberty acceleration, and induction of estrus [2,8-11]. Furthermore, genetic inactivation of the transient receptor potential channel Trpc2, the primary sensory ion channel of the VNO [12-14], results in a number of alterations in female reproductive behaviors, such as the absence of puberty acceleration, maternal aggression, and lordosis, and an increase in male-like sex behaviors [1,14-17]. However, experiments using either surgical VNO removal [18-21] or deletion of Trpc2 [13,14,16] led to a number of phenotypic discrepancies including unusual mounting levels toward males, and ultrasonic vocalizations and sex behavior toward females. Moreover, at least a portion of basal vomeronasal sensory neurons (VSNs) in Trpc2^-/-^ mice seem to retain some level of sensory responsiveness [11,22], possibly because of parallel signal amplification mechanisms [23-25], which makes Trpc2^-/-^ mice a much more complicated model to examine VNO-mediated function and behavior than previously anticipated.

The murine VNO sensory epithelium is segregated into at least two anatomically and molecularly distinct layers: apical VSNs express the G-protein Gαi2 and the V1R family of vomeronasal receptors, whereas basal VSNs express Gαo and members of the V2R receptor family [26-32]. The roles that these two VNO subsystems play in the control of innate, female-specific reproductive and sexual behaviors are largely unclear. Projections of these two VSN populations remain segregated at the level of the accessory olfactory bulb (AOB), and overlap only in more central brain areas, supporting both the possibility of divergent functions as well as synergy between the two subsystems. Several social and reproductive behaviors, such as aggression, puberty acceleration, and female attraction to males, have been suggested to be mediated by the basal VNO subsystem in some studies [3,22,33-35], and by the apical VNO in others [15,36-40]. To dissect VNO function in controlling female reproductive and neuroendocrine status, we have developed a mutant mouse strain harboring a conditional, olfactory marker protein (OMP) and Cre-mediated ablation of Gαo [41]. Cell-specific and time-dependent ablation of Gαo prevents these animals from detecting peptide and protein ligands by VSNs, leading to severe alterations in some social behaviors such as male-male and maternal aggression [41]. To fully understand the consequences of this conditional Gαo deletion, we undertook a considerably more comprehensive behavioral analysis of these mice and focused on the role of the basal Gαo vomeronasal subsystem in pheromone-stimulated sexual and reproductive behaviors of female mice. Our findings revealed that Gαo impacts on a much wider range of such behaviors than previously recognized, thus highlighting the importance of Gαo and the cells that express it in the control of pheromone-dependent sexual behaviors in female mice.

## Results

### Delayed puberty onset and defective puberty acceleration

**Figure 1:**
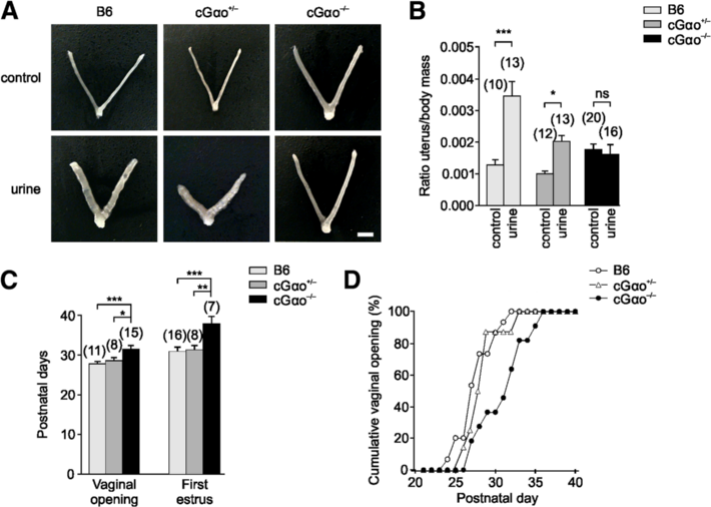
**Male urine fails to induce puberty acceleration in cGαo**^**-/- **^**females. (A,B)** Puberty acceleration is measured as uterine to body mass ratio increase after seven-day exposure to male urine. **(A)** Dissected uteri from 29-day old B6, cGαo^+/-a^and cGαo^-/-^ female mice unstimulated (top) and exposed to male urine (bottom). Scale bar, 5 mm. **(B)** Male urine exposure results in an increase in uterine mass in B6 and cGαo^+/-^ controls but not in cGαo^-/-^ females (analysis of variance (ANOVA): F_2,80_ = 5.4, *P* <0.01; LSD: ***P <0.001 (B6), *P <0.05 (cGαo^+/-^), non-significant (ns) *P* = 0.6 (cGαo^-/-^)). **(C)** Delayed puberty in cGαo^-/-^ females. Average vaginal opening (ANOVA: F_2,33_ = 7.34, *P* <0.005; LSD: ***P <0.001 (B6), *P <0.05 (cGαo^+/-^)) and time of the first estrus (ANOVA: F_2,30_ = 9.54, *P* < 0.005; LSD: ***P < 0.001 (B6), **P <0.005 (cGαo^+/-^)) were analyzed. No significant differences between B6 and cGαo^+/-^ controls were found (LSD: vaginal opening, *P* = 0.42; first estrus, *P* = 0.63). **(D)** Comparison of cumulative percentage of vaginal opening between the three genotypes. cGαo^-/-^ (black circles) mice needed three to four days longer than B6 (white circles) or cGαo^+/-^ (triangles) control mice to reach a 100% value. Number of tested animals per group is indicated in brackets.

A strategy to maximize reproductive success is the induction of estrus in females by their male partners. In juvenile female mice the entry into puberty is advanced after exposure to male urine, a phenomenon known as puberty acceleration or the Vandenbergh effect [6]. Puberty acceleration is strongly dependent on a functional VNO [8,9,15,19], and does not require an intact main olfactory epithelium (MOE) [42]. The pheromonal cues responsible for this effect have not been characterized with certainty, but small organic molecules have been proposed to have puberty-accelerating effects [15,37-39]. Some of these compounds are ligands that activate apical V1R/Gαi2-expressing VSNs [43]. Other studies implicate that major urinary proteins (MUPs), which are known to activate basal V2R/Gαo VSNs [22,41], or MUP-derived peptides accelerate puberty or contain ovulatory activity [33,34]. However, the effects of many of these molecules were difficult to reproduce by others [15]. It came as a surprise when we found that puberty acceleration - measured as stimulus-evoked uterine weight increase - was drastically reduced in the conditional, OMP/Cre Gαo-mutant (cGαo^-/-^) female mice versus heterozygous (cGαo^+/-^) and C57BL/6 (B6) controls (Figure 1A,B). We observed that stimulation with male urine failed to induce a significant increase in uterine to body mass ratio in cGαo^-/-^ females (least significant difference (LSD): *P* = 0.6) whereas B6 and cGαo^+/-^ control females exhibited a substantial gain after urine exposure (LSD: *P* <0.001 and <0.05, respectively) (Figure 1A,B). These results indicate that female puberty acceleration by cues present in male urine is severely reduced or absent in the mutant mice and that intact Gαo function is necessary to mediate pheromonal responses that increase uterine mass.

Previous studies showed that surgical VNO lesions also delayed the onset of puberty on grouped prepubertal females not exposed to male odors [9]. Therefore, we further characterized female juvenile mice to determine whether insensitivity to puberty accelerating cues is accompanied by a delay in puberty. Control B6, cGαo^+/-^, and cGαo^-/-^ females were examined for vaginal opening and first estrus. Vaginal opening was significantly delayed, by three to four days, in cGαo^-/-^ females compared with B6 and cGαo^+/-^ (B6: 27.7 ± 0.6; cGαo^+/-^: 28.6 ± 0.7; cGαo^-/-^: 31.5 ± 1.1; analysis of variance (ANOVA): F_2,33_ = 7.34, *P* <0.005; LSD: *P* <0.001 (B6), *P* <0.05 (cGαo^+/-^); Figure 1C,D). Similarly, the first estrus was also significantly delayed by approximately six days in cGαo^-/-v^ersus B6 females (B6: 30.9 ± 0.9; cGαo^+/-^: 31.3 ± 1.7; cGαo^-/-^: 37.4 ± 1.7; ANOVA: F_2,30_ = 9.54, *P* <0.005; LSD: *P* <0.001 (B6), *P* <0.005 (cGαo^+/-^); Figure 1D). No significant differences were found between B6 and cGαo^+/-^ controls (LSD: vaginal opening, *P* = 0.42; first estrus, *P* = 0.63). Thus, cGαo^-/-^ females displayed a delay in puberty that could not be altered by exposure to puberty-accelerating pheromones present in male urine.

### Altered estrus induction and estrous cycles

**Figure 2:**
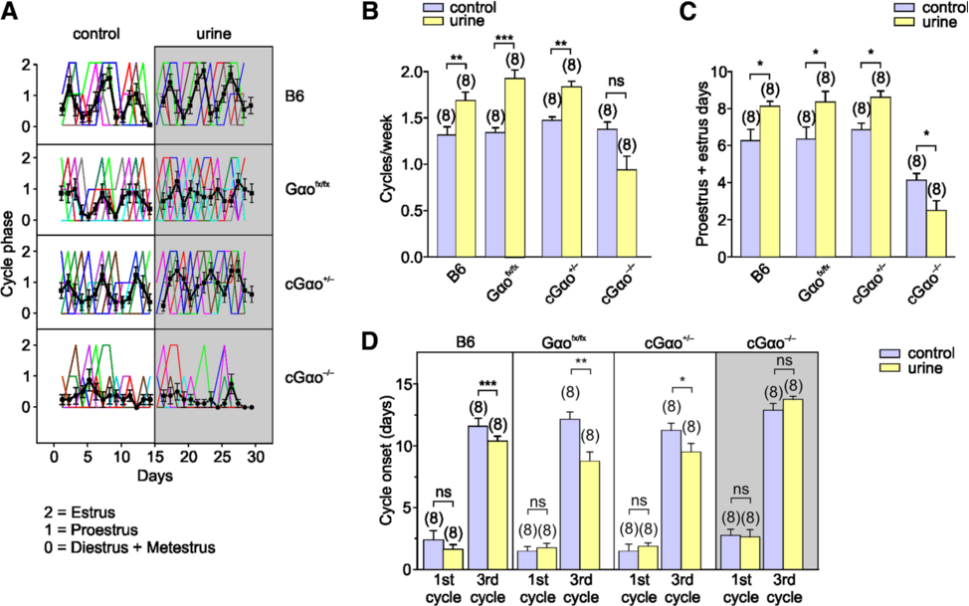
**cGαo**^**-/- **^**females show irregular estrous cycles that cannot be modified by male urine. (A)** Estrous cycles of group-housed cGαo^-/-^, Gαo^fx/fx^, cGαo^+/-^, and B6 female mice were monitored for two weeks under no stimulation (left) and two more weeks after male urine exposure (right, grey). Numbers on y-axis correspond to different estrous phases: 2, estrus; 1, proestrus; 0, diestrus and metestrus. Each color line represents individual subjects and black lines the average of all animals. A full cycle is calculated as the event between two estrous phases. In cases where no estrus is detected before return to diestrus/metestrus, the earliest proestrus phase is considered as the beginning/end of the cycle. **(B)** Male urine exposure during a two-week interval results in a cycle frequency increase in B6, Gαo^fx/fx^, and cGαo^+/-^ but not in cGαo^-/-^ mice (paired t-test: ***P < 0.001 (Gαo^fx/fx^), **P <0.005 (B6 and cGαo^+/-^), non-significant (ns) *P* = 0.06 (cGαo^-/-^)). **(C)** The number of receptive days (estrus and proestrus) after male urine exposure is increased in adult B6, Gαo^fx/fx^, and cGαo^+/-^ and reduced in cGαo^-/-^females (paired t-test: *P <0.05, for all strains). The number of days in estrus and proestrus is lower in cGαo^-/-^ when compared to control strains (analysis of variance: F_1,63_ = 7.83, *P* <0.01; least significant difference: *P* <0.001 in all cases; no significant difference is found between control genotypes: *P* = 0.27 to 0.71). **(D)** Two-week urine exposure induces an advancement of the third cycle by nearly two days in B6, Gαo^fx/fx^, and cGαo^+/-^ but not in cGαo^-/-^ females (paired t-test: ***P <0.001 (B6), **P <0.01 (Gαo^fx/fx^), *P <0.05 (cGαo^+/-^), ns *P* = 0.14 (cGαo^-/-^)).

To further explore Gαo-mediated effects on female reproductive fitness, we investigated the impact of chemosensory cues on the timing of behavioral estrus and ovulation in adult females. In this process, known as the Whitten effect [5], ovulation can be promoted in a group of adult female mice following exposure to urinary cues from adult males. To determine whether Gαo signaling is required for estrus induction, we analyzed the frequency and duration of the estrous cycles of group-housed cGαo^-/-^ mice before and after male urine exposure. We monitored the estrous cycles of cGαo^-/-^ mice and three different control strains: heterozygous cGαo^+/-^, ‘floxed’ Gαo mutants lacking Cre-recombinase (Gαo^fx/fx^), and B6 females. Each group was monitored during a four-week interval, in which we exposed the animals to adult male urine during the last two weeks (Figure 2A). B6, cGαo^+/-^, and Gαo^fx/fx^ control mice displayed regular and consistent estrous cycles before and after exposure to male urine (Figure 2A). However, cGαo^-/-^ females exhibited irregular cycles with a decreased number of receptive days (estrus or proestrus) (Figure 2A,C). After exposure to male urine, estrous cycles of B6, cGαo^+/-^, and Gαo^fx/fx^ mice showed a significant increase in cycle frequency (paired t-test: *P* <0.005 (B6), <0.001 (cGαo^+/-^), <0.005 (Gαo^fx/fx^); Figure 2B) and in the number of receptive days (estrus and proestrus) (paired t-test: *P* <0.01 in all cases; Figure 2C). By contrast, estrous cycles of cGαo^-/-^ females following male urine exposure did not increase in cycle frequency (Figure 2B) or receptive days (Figure 2C). Interestingly, Gαo ablation also seemed to cause a reduction of days in estrus and proestrus even without urine stimulation (ANOVA: F_1,63_ = 7.83, *P* <0.01; LSD: *P* <0.001 in all cases when compared to B6, Gαo^fx/fx^, and cGαo^+/-^; no difference occurred between control genotypes: *P* = 0.27 to 0.71; Figure 2C). Exposure to male urine also resulted in a significant cycle acceleration of nearly two days after three estrous cycles monitored in B6 females (paired t-test: *P* <0.001 (B6), *P* <0.05 (cGαo^+/-^), *P* <0.01 (Gαo^fx/fx^)), whereas there was no significant change in estrous cycle onset in cGαo^-/-^ females after the male urine two-week stimulation (paired t-test: *P* = 0.14; Figure 2D). cGαo^-/-^ females showed lower cycle frequency rate (paired t-test: *P* = 0.06) and number of receptive days (paired t-test: *P* <0.05) after male urine exposure (Figure 2B,C), suggesting that sensory input to male cues may not be completely abolished in these mice. Taken together, these findings revealed that Gαo is a necessary requirement in female mice for the display of sexual receptivity, reproductive status, and physiological changes promoted by male urinary pheromones.

### Reproductive performance and sex hormone levels

**Figure 3:**
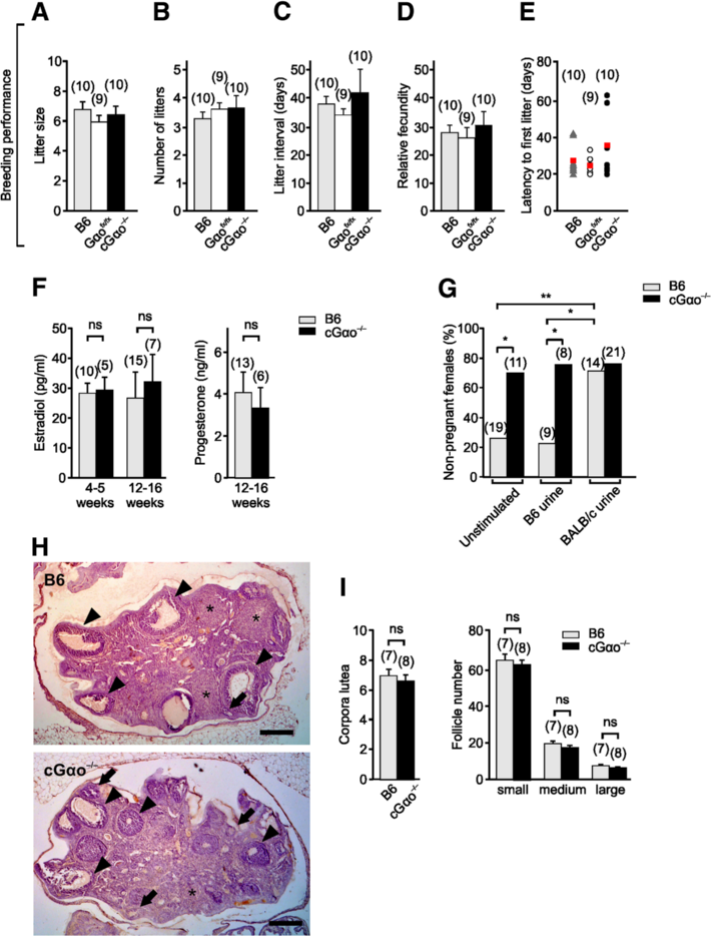
**Analysis of ovary morphology, steroid hormone levels, reproductive performance, and Bruce effect test. (A-E)** Normal breeding performance in cGαo^-/-^ females during a four-month interval, analyzed as litter size **(A)**, number of litters **(B)**, litter interval **(C)**, relative fecundity **(D)**, and latency to first litter **(E)** (analysis of variance (ANOVA): F_2,26_ = 0.44 to 0.95, *P* = 0.40 to 0.65). Circles/triangles in E represent individual subjects, red squares indicate mean values. **(F)** Plasma estradiol and progesterone levels at proestrus did not differ between B6 control and cGαo^-/-^ mice measured in juveniles (4 to 5 weeks) and adults (12 to 16 weeks) (t-test: *P* = 0.61 (juveniles) and 0.2 (adults) for estradiol; *P* = 0.68 for progesterone). **(G)** Near-maximum levels of non-pregnant females are observed in cGαo^-/-^ females in the Bruce effect assay when tested with familiar urine or under unstimulated conditions (ANOVA: F_2,78_ = 6.52, least significant difference (LSD): *P <0.05). B6 females perform normally and discriminate familiar versus unfamiliar urine cues (LSD: *P <0.05, **P <0.01). **(H)** Histological sections of ovaries from both adult B6 control (top) and cGαo^-/-^ mice (bottom) show the presence of follicles with antrum at different stages of folliculogenesis (arrows indicate small- or medium-sized follicles arrowheads indicate large follicles ) as well as of corpora lutea (*). Scale bar, 250 μm. **(I)** Number of corpora lutea and follicles with antrum did not differ between B6 and cGαo^-/-^ mice (t-test: *P* = 0.18 to 0.56). Follicles were divided into three categories according to their size: 100 to 199 μm (small), 200 to 299 μm (medium), >300 μm (large or preovulatory). ns, non-significant.

Our finding that cGαo^-/-^ mice had fewer days in estrus and proestrus and less-regular estrous cycles was not caused by a general disruption of reproductive capacity in these mice. We compared several fertility parameters in B6, Gαo^fx/fx^ controls, and cGαo^-/-^ mice in continuous mating conditions over four months. cGαo^-/-^, Gαo^fx/fx^, and B6 females did not differ in litter size, average number of litters, litter interval, relative fecundity, or latency to first litter (ANOVA: F_2,26_ = 0.44 to 0.95, *P* = 0.40 to 0.65; Figure 3A-E). Consistent with these results, no obvious changes in ovary morphology were observed in cGαo^-/-^ females (Figure 3H). Follicle size (follicles with antrum: from very small to large, pre-ovulatory follicles) as well as the presence of corpus luteum in both genotypes revealed no differences in morphology and number (t-test: *P* = 0.18 to 0.56; Figure 3I). Furthermore, there was no significant difference in the levels of circulating estradiol at proestrus stage in both juvenile (4 to 5 weeks) and adult (12 to 16 weeks) females and progesterone in adults (t-test: *P* = 0.61, 0.20, and 0.68, respectively; Figure 3F), thus confirming that impaired behavioral and physiological responses in cGαo^-/-^ females did not result from an indirect effect of the mutation at the level of the ovaries.

### Bruce effect

We examined the Bruce effect, a form of olfactory imprinting that leads to pregnancy failure and depends on the formation and maintenance of a pheromonal recognition memory. In this process, recently mated female mice experience high pregnancy failure rates and return to estrus when exposed to unfamiliar male odors during a three- to four-day critical period of embryo implantation [44]. This pregnancy block requires an intact VNO [45,46]. The low molecular weight fractions of unfamiliar urine [47] and major histocompatibility complex (MHC) peptides of disparate MHC haplotypes [48] can induce the Bruce effect. Given that Gαo is essential for sensing of MHC peptides by VSNs [41], we asked whether pregnancy can still be terminated by unfamiliar urinary cues in cGαo^-/-^ mice. Females were exposed to urine from unfamiliar (BALB/c) males after a short 24 h mating period with B6 (familiar) males. The B6 females performed as expected: high pregnancy rates were observed when no further stimuli were applied (unstimulated) or when the mice were exposed to familiar B6 urine, and exposure to unfamiliar urine induced a low pregnancy rate (77% non-pregnant females) in B6 (Figure 3G). By contrast, cGαo^-/-^ females already showed near-maximum non-pregnancy rates under unstimulated conditions or after exposure to familiar urine, and stimulation with unfamiliar urine did not further increase these high failure rates (Figure 3G).

### Defective lordosis behavior

**Figure 4:**
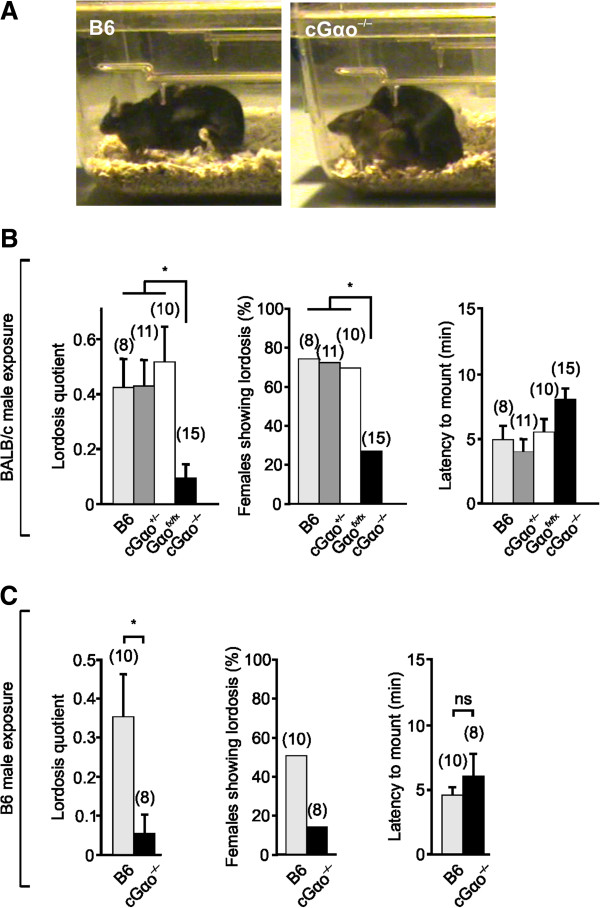
**Defective lordosis behavior in cGαo**^**-/- **^**females. (A-C)** Lordosis, a sexual stance that denotes female receptivity **(A)**, is severely reduced in cGαo^-/-^ females exposed to both BALB/c **(B)** and B6 **(C)** males. A reduction is found in the lordosis quotient using both B6 (t-test: *P* <0.05) and BALB/c males towards cGαo^-/-^ females (analysis of variance (ANOVA): F_3,44_ = 5.05; least significant difference (LSD): *P* <0.05), the percentage of females showing lordosis (ANOVA: F_3,44_ = 3.44; LSD: *P* <0.05), but not in the latency to mount females using both B6 and BALB/c stud males (*P* = 0.33 to 0.47). ns, non-significant.

We investigated pheromone-dependent, sexually receptive behaviors in B6 versus cGαo^-/-^ females (Figure 4). In cGαo^-/-^ females that were exposed to a sexually experienced control B6 male, we quantified lordosis, a female sexual stance in response to male mounting that denotes sexual receptivity and allows for successful copulation. Lordosis requires intact VNO-AOB function [1,2,21], and activation of the vomeronasal Vmn2r116 receptor by exocrine-gland-secreted peptide ESP1 enhances lordosis in mice [1]. Given that females deficient for Vmn2r116 show a striking deficit in lordosis [1] and that ESP1 detection is severely reduced in VSNs lacking Gαo [41], the display of lordosis should also be affected in cGαo^-/-^ females. Females were exposed to males from two different strains (B6 and BALB/c). These strains differ on the expression of ESP1: B6 males do not secrete ESP1 whereas BALB/c males do [1]. In both cases, we found a dramatic (four- to seven-fold) reduction in lordosis quotient (number of lordosis stances in response to male mounts) in cGαo^-/-^ mice versus B6, cGαo^+/-^, and Gαo^fx/fx^ controls (t-test: *P* <0.05 (B6 males); ANOVA: F_3,44_ = 5.05; LSD: *P* <0.01 (BALB/c males) ). B6, Gαo^fx/fx^, and cGαo^+/-^ controls did not differ statistically (*P* = 0.51 to 0.99). We also observed a strongly reduced number of females showing lordosis (three- to five-fold reduction) in cGαo^-/-^ mice versus B6, cGαo^+/-^, and Gαo^fx/fx^ controls (ANOVA: F_3,44_ = 3.44; LSD: *P* <0.05; Figure 4B,C). The overall latency of cGαo^-/-^ females to be mounted by males in the lordosis assay was similar in all cases (Figure 4B,C). Thus, Gαo played a critical role in the display of sexual receptivity and the detection of lordosis-enhancing pheromones by female mice.

### No display of male-typical mating behaviors in Gαo-mutant females

**Figure 5:**
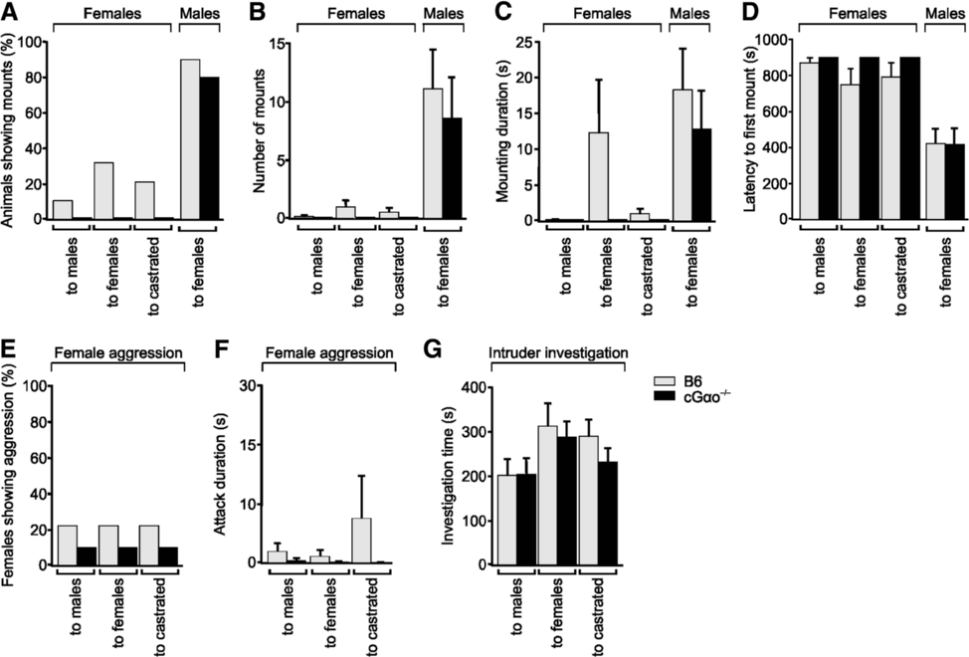
**No display of male-typical behaviors in cGαo**^**-/- **^**females. (A-D)** B6 and cGαo^-/-^ females display little or no mounting behavior toward male, female, or castrated male intruders (analysis of variance (ANOVA): F_1,56_ = 3.339 to 6.286, *P* = 0.06 to 0.88). B6 and cGαo^-/-^ male mice show similar mounting behaviors toward female intruders (t-test: *P* = 0.49 to 0.96). **(E,F)** Aggression levels toward males remain low in both B6 and cGαo^-/-^ female mice (ANOVA: F_1,56_ = 2.182, *P* = 0.15 (attack duration)). **(G)** There are similar intruder investigation times for both B6 and cGαo^-/-^ female mice (ANOVA: F_1,56_ = 0.653, *P* = 0.42).

Lack of receptivity to males could be a consequence of defective gender discrimination. Some studies have argued that an intact main olfactory system, but not VNO, is required for gender discrimination given that mice after surgical removal of the VNO or AOB are still able to distinguish between male and female urinary volatiles [20,21]. Other investigations concluded that it is the VNO that mediates sex discrimination [13,16]. In these latter experiments, Trpc2^-/-^ mice of both sexes displayed an increase in mounting behavior toward any conspecific regardless of the gender, and a reduction in female-typical behaviors such as maternal aggression and nesting [13,14,16]. It is unclear whether these alterations in behavior are a consequence of the existence of VNO-dependent neural circuits underlying gender-specific behaviors [16] or, alternatively, whether those behaviors are caused by hormonal changes [21]. In addition, ablation of Gαo in female mice results in defective maternal aggression [41]. These findings prompted us to test whether conditional Gαo deletion has a crucial effect on gender recognition and sex-specific behaviors (Figure 5). We quantified mounting behavior of cGαo^-/-^ female residents to either male, female, or castrated male intruders that were independently introduced into the resident’s home cage during a 15 min test period. None of the cGαo^-/-^ females tested exhibited mounting to any of the intruders, although some low levels of mounting (measured as number of mounts, mounting duration, and latency to first mount) were observed in 10% to 30% of the control B6 females, especially to female and castrated intruders (Figure 5A-D). Other types of male-typical behaviors such as territorial aggression, quantified as the number of aggressive animals or attack duration, remained low in both cGαo^-/-^ and B6 control female mice (ANOVA: F_1,56_ = 2.182, *P* = 0.15; Figure 5E,F). To test whether the lack of mounting in the cGαo^-/-^ mice was a consequence of reduced social interaction with the intruder, we measured sniffing times of the resident females to the intruder (Figure 5G). No significant difference in sniffing duration was found in cGαo^-/-^ females versus B6 controls (ANOVA: F_1,56_ = 0.653, *P* = 0.42; Figure 5G). Thus, deletion of Gαo did not enhance the display of male-specific behaviors in female mice. Rather, there seems to be a repression of mounting in cGαo^-/-^ females. To investigate whether repression of sexual behavior is a widespread condition in cGαo^-/-^ animals, we measured mounting towards female mice in cGαo^-/-^ males. We introduced an estrous female in the home cage of sexually naïve cGαo^-/-^ male residents and found high levels of all measures of mounting, including mounting animals, number of mounts per animal, mounting duration, and latency to first mount, that were not significantly different from those of the B6 control males (t-test: *P* = 0.49 to 0.96; Figure 5A-D). Thus, unlike Trpc2 mutants, cGαo^-/-^ males did not exhibit obvious deficits in male-female choice of sexual partner.

**Figure 6:**
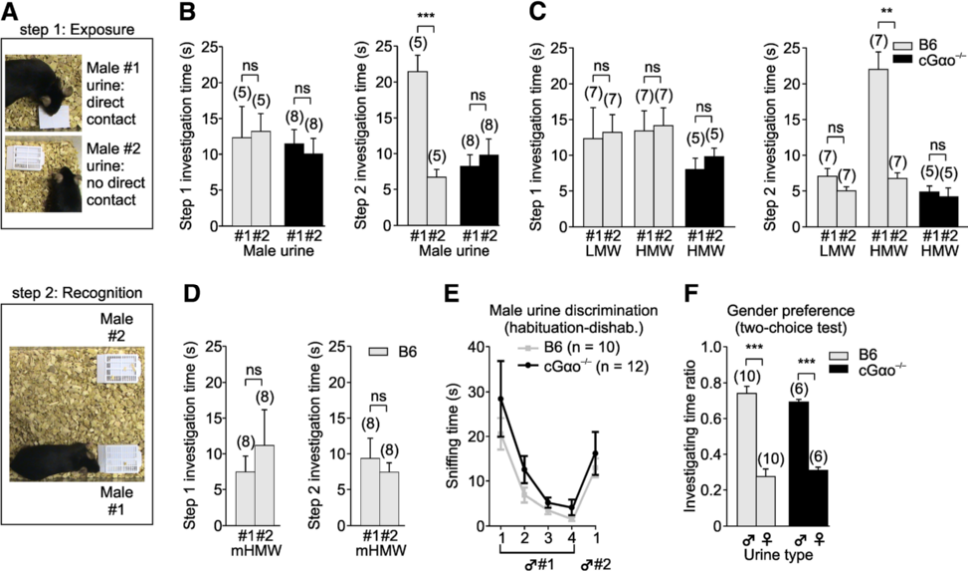
**cGαo**^**-/- **^**females are not attracted to familiar male urine in a two-choice ownership recognition test. (A)** To evaluate attraction and ownership recognition, a two-step experiment is performed: in an identity-learning phase (step 1: exposure), females are exposed to filter papers with urine streaks from two B6 males, one allowing direct physical contact (male #1 urine) and a second male urine (male #2) deposited on a filter paper in a meshed plastic cassette to prevent direct physical contact. In the recognition phase (step 2: recognition), the same females are given a choice between male #1 and #2 urine volatiles (no direct contact). **(B)** Preference for the previously contacted urine displayed by B6 females is absent in cGαo^-/-^ female mice in step 2 (t-test: ***P <0.001 (B6); non-significant (ns) *P* = 0.19 (cGαo^-/-^)). **(C)** Preference in control B6 females is induced only by high molecular weight (HMW, >10 kDa) fraction of male urine in step 2 (t-test: *P* = 0.17 (low molecular weight (LMW)), **P <0.01 (HMW)). HMW activity is lost when tested on cGαo^-/-^ females (t-test: *P* = 0.59). **(D)** HMW fraction is incubated with menadione, a competitive displacer that releases hydrophobic small volatile molecules from the major urinary proteins β-barrel binding pocket. Menadione-incubated HMW (mHMW) fraction prevents the formation of a preference in step 2 in wild-type animals (t-test: *P* = 0.54). **(E)** Both B6 and cGαo^-/-^ females are able to discriminate urine volatiles from two different B6 males in a habituation-dishabituation paradigm (analysis of variance (ANOVA): F_1,97_ = 2.77, *P* = 0.1; least significant difference (LSD): *P* <0.005). **(F)** Preference for male versus female urine volatiles in a two-choice preference test is maintained intact in cGαo^-/-^ females (ANOVA: F_1,31_ < 0.001, *P* = 1 ; LSD: ***P <0.001). ns, non-significant.

To further assess whether cGαo^-/-^ mice are able to discriminate between odors of different genders, we performed a simultaneous odor choice test (see Methods) for urinary odors in cGαo^-/-^versus B6 female mice. Following odor presentation, both groups spent more time investigating intact male urine versus female urine (LSD: *P* <0.001 (cGαo^-/-^), *P* <0.001 (B6); Figure 6F). This indicates that cGαo^-/-^ females are capable of discriminating urinary odors of males versus females, presumably after their detection by the MOE. Therefore, both cGαo^-/-^ males and females were able to discriminate gender cues present in urine and did not exhibit enhanced sexual-specific behaviors such as indiscriminate male and female mounting.

### Loss of attraction to familiar ownership signals

Under natural conditions, sexual attraction to the scent of dominant males may provide females with benefits in terms of proper mate choice and pregnancy onset and maintenance [35,49]. The ability to detect dominant males requires females to discriminate between different potential mating partners and, subsequently, display a preference for one of them. Female mice are attracted to and show a preference for airborne urinary volatiles from familiar individual males whose scent marks they have previously contacted physically relative to males whose scent is unfamiliar [3,4,50]. To determine whether this preference requires Gαo-dependent vomeronasal chemoreception, during an identity-learning phase (Figure 6A, step 1: exposure), female mice were presented with urine streaks from two unrelated B6 males, each singly housed (see Methods). We placed urine from the first male on a filter paper allowing direct physical contact with the odor source whereas urine from the second male was deposited on filter paper that was placed in a meshed plastic box [51], thus preventing direct physical contact. At this point, females showed no innate preference for any of the tested urine sources (Figure 6B, step 1). In a subsequent recognition phase (Figure 6A, step 2: recognition), the females were then given a choice between urine scents from the two males under conditions that precluded direct physical access to both urine sources. We measured overall investigation times during a 5 min test period, that is, the time spent in close proximity to the plastic box including pulling and gnawing at the box and attempting to access the stimulus. In B6 females, there was a three-fold preference for the urine they had previously encountered by physical contact (t-test: *P* <0.001; Figure 6B, step 2). By contrast, this preference was absent in cGαo^-/-^ female mice (t-test: *P* = 0.19) and investigation times for both stimuli remained relatively low (Figure 6B, step 2), indicating that experience with both volatile and nonvolatile components of male urine failed to induce familiar male preference and attraction.

To determine potential urinary ligands underlying this type of scent recognition by female mice, we repeated this test using fractionated male urine in order to separate larger MUPs from small molecules such as urinary peptides. Behavioral analysis revealed that the MUP-containing high molecular weight (HMW; >10 kDa) fraction of male urine was able to induce a preference in control B6 females, whereas the low molecular weight fraction (LMW; <10 kDa) showed no significant activity (t-test: *P* = 0.17 (LMW); *P* <0.01 (HMW); Figure 6C). The HMW-dependent activity was lost when tested on cGαo^-/-^ females (t-test: *P* = 0.59; Figure 6C). MUPs present in HMW are nonvolatile proteins of around 20 kDa in size and can only be detected when physical contact is allowed. However, MUPs fold into a β-barrel structure that binds small hydrophobic volatile molecules that are released into the air and can be detected without physical contact. Competitive displacement of these ligands, by incubation of the HMW fraction with menadione [22,52], blocks volatile release and virtually transforms the HMW fraction odorless if physical contact is inhibited. Incubation of the HMW fraction with menadione prior to assaying for behavior prevented the formation of a preference for any of the urine sources in wild-type animals (t-test: *P* = 0.54; Figure 6D), indicating that associative learning of nonvolatile HMW fraction with volatiles is necessary to generate the preference.

Collectively, these results indicate that Gαo is required to develop a learned association with a nonvolatile ownership signal and that vomeronasal MUP detection is likely to mediate this effect. To verify that such ownership recognition requires scents to be associated with nonvolatile identity signatures and to confirm that cGαo^-/-^ mice were not anosmic, we evaluated their ability to discriminate volatile odors from two different males. Using a habituation-dishabituation paradigm [41,53,54], we found that both B6 and cGαo^-/-^ females were able to discriminate urine volatiles from different male individuals (t-test: *P* <0.05 (cGαo^-/-^), *P* <0.001 (B6; Figure 6E), indicating an intact MOE function. This result is consistent with previous observations showing that surgical removal of the VNO does not affect the ability to distinguish urinary odors from different males [20]. Furthermore, cGαo^-/-^ females displayed intact preference for male versus female urine in a two-choice preference test (LSD: *P* <0.001 (cGαo^-/-^), *P* <0.001 (B6); Figure 6F), also shown to remain unaffected after VNO or AOB surgical removal [20,21].

### No impact of the conditional Gαo deletion on Gαo expression in reproductive central nervous system nuclei

**Figure 7:**
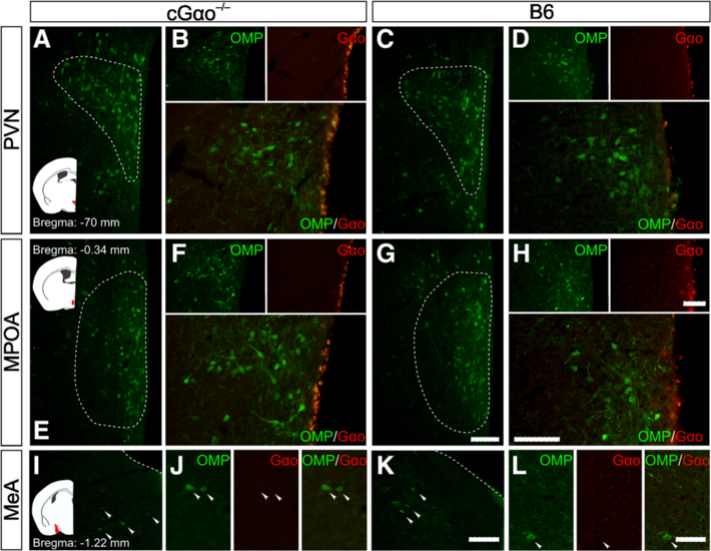
**No impact of the conditional Gαo deletion on Gαo expression in reproductive central nervous system nuclei. (A-L)** Representative olfactory marker protein (OMP) immunohistochemistry in the paraventricular nucleus and medial preoptic area of the hypothalamus as well as the medial amygdala in B6 and cGαo^-/-^ female mice. Schematics on the left indicate the studied brain areas (red). Double immunostaining for OMP (arrowheads) and Gαo in cGαo^-/-^**(B,F,J)** and B6 females **(D,H,L)** in the same brain areas reveal no overlap between both signals. Scale bar, 100 μm **(A-K)** and 50 μm **(J,L)**.

Scent ownership recognition and preference, sexual receptivity, and estrous cycle regulation all require intact connections between the primary olfactory regions and central limbic and hypothalamic regions [50]. Such brain regions are known to contain neurons positive for OMP [55,56]. Therefore, as a further control and to verify that the described loss of functions by the conditional Gαo ablation were indeed caused by a loss of signaling in VSNs, not in neurons of the central nervous system (CNS), we performed double-label immunostaining for OMP and Gαo in the CNS. Consistent with previous observations [55,56], we found OMP-positive cells in hypothalamic and amygdaloid nuclei of B6 and cGαo^-/-^ females, including the paraventricular nucleus (PVN), medial preoptic area (MPOA), and medial amygdala (MeA) (Figure 7). Importantly, however, we found no overlap between OMP and Gαo immunostaining in any of these regions (Figure 7B,D,F,H,J,L). Moreover, both OMP and Gαo staining in th PVN, MPOA, and MeA of cGαo^-/-^ mice revealed very similar patterns of protein expression compared to B6 controls (Figure 7A-L).

**Figure 8:**
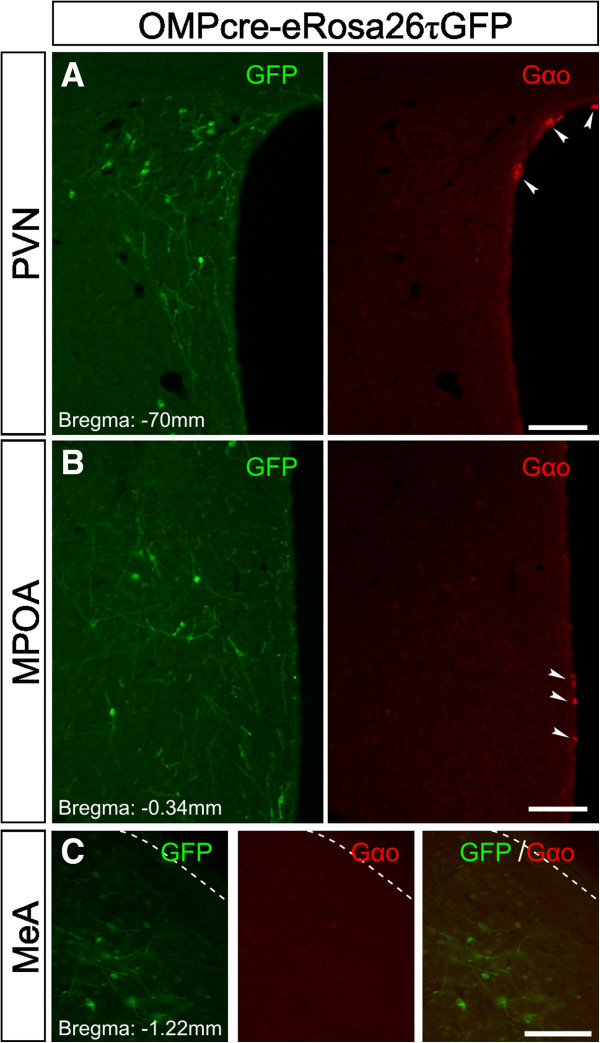
**GFP-positive central nervous system cells of an olfactory marker protein-τGFP reporter mouse line.** Double immunostaining for GFP (left panels; green) and Gαo (right panels; red) in brain slices of reporter mouse line OMPCre-eRosa26τGFP. In these mice, olfactory marker protein (OMP) expression enables Cre-mediated recombination of a stop codon preceding the τGFP locus and therefore driving continuous expression of GFP in all OMP-positive cells. GFP staining can be found in the paraventricular nucleus **(A)**, medial preoptic area **(B)**, and medial amygdala **(C)** of adult female mice. Double staining showed no overlap between GFP and Gαo (arrowheads). Scale bar, 100 μm. GFP, green fluorescent protein.

In a different approach, we also examined the reporter mouse line OMPCre-eRosa26τGFP in which OMP expression drives the continuous expression of tau:green fluorescent protein fusion (τGFP). As expected, we found GFP expression in the PVN, MPOA, and MeA of adult female mice (Figure 8). Double staining of GFP with Gαo antibody in this mouse also revealed no overlap between these two markers in any of the regions examined (Figure 8A-C). Hence conditional, OMP-Cre-mediated ablation of Gαo is unlikely to affect the function of OMP-expressing cells in these brain nuclei.

## Discussion

The past decade has seen rapid progress in deciphering the essential role of the mammalian accessory olfactory system in chemical communication and the regulation of social behaviors [12,57], but the functional significance of the anatomical and molecular segregation into VSNs that express either of two different G protein subunits, Gαo or Gαi2, has not been resolved. Elucidating the specific behavioral roles of each of these VSN populations is required to understand whether and how these subsystems work together to represent the sensory environment and how exactly they control behavioral responses [7].

To address these questions, we have developed a mouse strain that harbors a conditional deletion of the *Gnao1* gene under the control of the promoter of the *Omp* gene [41]. We have shown that behavioral responses that depend on a functional main olfactory system are normal in these mice and that the mutation has no impact on Gαo expression in OMP-positive neurons that are present in some reproductive CNS brain nuclei. We observed no obvious changes in the amount and distribution of OMP-positive cells in hypothalamic and amygdaloid nuclei in Gαo mutants and there was no obvious overlap between Gαo and OMP immunoreactivity in both B6 mice and a mouse line expressing an OMPCre-eRosa26τGFP reporter. Furthermore, blood estradiol and progesterone levels, ovary morphology, and general fertility parameters were all normal in the Gαo mutants. Thus, our conditional Gαo-mutant mice constitute an appropriate model to examine the role of Gαo-expressing VSNs in pheromone-stimulated behavioral responses of female mice. Deletion of critical signaling molecules such as Gαo, Trpc2, and Gγ8 causes a significant reduction in the number of basal VNO neurons [13,41,58]. Given that we do not know whether this cell loss reflects loss of specific V2R-expressing VSNs, we cannot fully rule out the possibility that some of the phenotypes identified here reflect dominant or neomorphic phenotypes. Nonetheless, our results clearly define important functions of Gαo-expressing VSNs in different behavioral repertoires.

A key result of this report is that Gαo signaling impacts on a much wider range of pheromone-dependent behaviors than previously anticipated. For example, our results reveal an unexpected delay in the initiation of puberty (Figure 1C,D) and an altered estrous cycle (Figure 2) in the mutant mice, even without active stimulus presentation. This suggests that sensory input via Gαo-expressing VSNs is required for the normal display of puberty onset and the regulation of ovulatory signals [9]. Furthermore, selective ablation of Gαo conferred insensitivity to male urinary pheromones that facilitate the display of female reproductive behaviors: urine-stimulated puberty acceleration was defective in Gαo-mutant females (Figure 1A,B). The chemical nature of the puberty-accelerating pheromone(s) is still unclear but at least two reports have linked puberty acceleration to MUPs or MUP-derived peptides [33,34]. MUPs activate basal V2R-positive VSNs and their VNO detection is lost following Gαo deletion [22,41]. Thus, these studies are consistent with our findings showing defective puberty acceleration in Gαo mutants. On the other hand, several reports have indicated a role for small organic molecules in puberty acceleration [15,37-39] and some of these molecules are known to activate apical V1R/Gαi2-expressing VSNs which function normally in the Gαo mutants [41,43]. One possible explanation for these seemingly divergent results is that Gαo- and Gαi2-expressing subsets of VSNs could both be involved in these effects. Such a scenario is not without precedent: the display of male territorial aggression and maternal aggression also seems to depend on the activation of both Gαi2- and Gαo-expressing VSNs [36,41,59]. Besides a lack of effect of male urine to induce uterine growth, we observed that Gαo-mutant females showed larger uteri in the absence of stimulation (Figure 1B). One potential explanation for this result is that the basal VSNs are required for the Lee-Boot effect [60] in which female urine may suppress uterine maturation. If so, in the absence of suppression, uterine weight will increase regardless of the stimulation. However, Gαo mutants displayed a delayed first estrus (Figure 1C) and unstimulated adults did not show more frequent estrous cycles (Figure 2), as would be expected in mice deficient for the Lee-Boot effect. Therefore, we cannot currently confirm a direct dependency of this effect on Gαo signaling.

Another surprising finding was the critical role of Gαo signaling in pheromone-induced estrus induction in adult mice. Interestingly, Gαo ablation not only abolished male urine-induced estrus induction, but also seemed to cause a reduction of days in estrus and proestrus (Figure 2C). This result suggests that Gαo-mutant females are not entirely unresponsive to estrus-modifying pheromones but the functional outcome of such chemosignals is altered, perhaps as a result of defective processing or integration with other pheromonal cues. Consistent with this possibility, small organic molecules such as 2-sec-butyl-4,5-dihydrothiazole, dehydro-exo-brevicomin, and α- and β-farnesenes have estrus-inducing effects in mice [61,62]. These cues are present in male urine, are known to activate VSNs of the apical VNO neuroepithelium [43], and thus should still be detectable in the absence of Gαo.

Conditional deletion of Gαo also has severe consequences on female sexual receptivity, that is, lordosis behavior. Two measures of lordosis, lordosis quotient and number of females showing lordosis, indicated that this pheromone-stimulated behavioral response was absent or strongly diminished in the mutant mice (Figure 4). Thus, intact Gαo signaling is essential for this innate, female-specific sexual display. These results are consistent with studies demonstrating that the *Vmn2r116* receptor is involved in lordosis behavior [1] and that detection of ESP1 is severely reduced in VSNs lacking Gαo [41]. The fact that lordosis induced by exposure to both B6 and BALB/c mice was diminished in the Gαo mutants indicates that, besides ESP1 and *Vmn2r116*, other pheromones and V2R receptors are probably involved in this behavior because B6 mice do not secrete ESP1 [1]. We cannot yet completely rule out that the cycling phenotype as observed here impacts on lordosis but, as there was no evidence for ovarian or hormonal imbalance in our experiments (Figure 3), major effects of the cycling phenotype on lordosis seem unlikely.

Importantly, Gαo-mutant females were not only defective in a variety of pheromone-stimulated innate behaviors but also in learned social responses to pheromones. Employing an established paradigm to assess mate recognition (Figure 6), our results provide direct evidence in support of a model in which Gαo-positive VSNs are critically involved in the detection of molecular cues related to genomic individuality. Scent ownership recognition experiments demonstrated directly that this test required contact to chemical cues present in the HMW fraction of urine (Figure 6C,D), consistent with a proposed role for MUPs in this function [3,35]. Preference for individual male scents requires an associative learning step to provide a linkage between information contained in the volatile and the nonvolatile HMW urinary fractions; we demonstrated here that this learning requires intact Gαo signaling (Figure 6B-D). Of note, Gαo-mutant females could still discriminate the urine of two different males in a habituation-dishabituation test (Figure 6E) and showed a preference for male versus female urine in a two-choice test (Figure 6F), indicating that olfactory discrimination abilities were normal in these mice. Furthermore, defective scent ownership recognition was not due to a loss of gender discrimination: Gαo-mutant females did not display male-typical mating behaviors toward other conspecifics (Figure 5). Such indiscriminate mounting has been reported previously in mice deficient in the cation channel Trpc2 [13,14,16]. One of these studies [16] employed a large open arena, but it is unclear whether the behavioral apparatus impacts on the display of male-like behaviors in Trpc2 mutants.

We were unable to observe a second pheromone-dependent learning paradigm, the Bruce effect, in Gαo-mutant females. Near-maximum non-pregnancy rates occurred with exposure to familiar cues or even without any additional stimulus exposure. We cannot yet determine whether this reflects a failure of the mutant mice to discriminate familiar from unfamiliar cues or whether other deficits such as poor mating performance (lordosis), shorter receptive periods (estrus), and loss of mate recognition capabilities influence the outcome of this test. Most likely, the low pregnancy rates reflect a combination of all of these defects. Remarkably, cGαo^-/-^ mice exhibited high variability on first litter latency, eventually expanding to values of up to 60 days (Figure 3E), which could be consistent with a potential fertility defect. However, on average, first litter latencies and other fertility values in the null mice were not significantly different from the controls. As an explanation for the apparent contradiction between low Bruce effect performance and normal fertility parameters, we believe that the sum of the described reproductive deficiencies may remain unnoticed in a laboratory environment: during the Bruce effect test, males and females are mated for just 24 h in contrast to the fertility monitoring in which breeding pairs remain in permanent contact. However, Gαo-mutant females would be unlikely to stay competitive under natural conditions where animals are subject to time-limited sexual encounters and where optimal reproductive performance is essential for reproductive success.

## Conclusion

Our experiments provide a systematic analysis of the importance of Gαo and the Gαo-expressing VSN subsystem for pheromone-stimulated sexual behaviors of female mice. We can now begin to determine the extent to which apical and basal VNO subsystems regulate functionally distinct behavioral repertoires. Future studies targeting specific pheromone receptor classes should also help to elucidate the neuronal logic underlying vital sexual and mating behaviors.

## Methods

### Mice

Animal care and experimental procedures were performed in accordance with the guidelines established by the animal welfare committee of the University of Saarland. Mice were kept under standard light/dark cycle with food and water *ad libitum. Gnao1*-deficient mice were generated as described [41]. Briefly, floxed *Gnao1* (denoted as Gαo^fx/fx^) mice were crossed with mice carrying a transgene directing the expression of *Cre* recombinase under the control of the OMP promoter (OMP-Cre mice; B6;129P2-*Omp*^*tm4(cre)Mom*^/MomJ) [63]. OMP is an abundant cytosolic protein expressed by all mature olfactory sensory neurons and VSNs. More breeding established offspring that were homozygous for the floxed *Gnao1* alleles and heterozygous for *Cre* and *OMP* (Gαo ^-/-^OMP-Cre^+/-^Or cGαo^-/-^). In these mice, Cre-mediated *Gnao1* deletion was restricted to OMP-positive cells. Heterozygous littermates for floxed *Gnao1* (Gαo^+/-^OMP-Cre^+/-^, denoted as cGαo^+/-^), Gαo^fx/fx^ not crossed with the OMP-Cre line, and C57BL/6 mice (denoted as B6) were used as controls. The background of the Gαo^fx/fx^ is pure 129SV agouti. The OMPCre-eRosa26τGFP mice were generated by crossing OMP-Cre mice with eR26-τGFP reporter mice [64] to express τGFP in all OMP-expressing neurons. Following Cre-mediated excision of a stop sequence, the reporter mice express a fusion protein of the microtubule-associated tau protein with GFP (τGFP) [65] in the ROSA26 locus [66].

### Urine fractionation

Urine was freshly collected, pooled from more than three B6 males (8 to 12 weeks old, sexually naïve) and size fractionated by centrifugation (14,000 g, 30 min) using Microcon 10 kDa molecular weight cut-off ultrafiltration columns (Millipore, Schwalbach, Germany). The first flowthrough was collected as the LMW fraction. To obtain the HMW fraction containing MUPs purified from urine, the centrifugation retentate was washed with one volume of PBS three times and re-concentrated to reach the same initial concentration of urine. HMW displacement of small ligands with menadione was performed as described [41,52]. Briefly, the HMW fraction was incubated with menadione dissolved in ethanol (4 mg/ml) in a 1/10 proportion (1 part of menadione for 10 parts of solution), for 30 min. Then, the solution was washed two times with PBS.

### Female estrous cycle

Female estrous cycle was determined by observation of vaginal cytological extracts obtained by flushes of PBS gently applied with a glass Pasteur pipette on the external genital opening. This method avoids pseudopregnancy inducible by mechanical stimulation [67].

### Reproductive performance

An assay to determine the reproductive performance was used to calculate the number of litters, litter size, litter interval, relative fecundity, and latency to first litter. Females 5 to 12 weeks old were used and measured for a duration of four months. Breeding started with both males and females being sexually inexperienced. The male was kept in the cage for the duration of the test. Mutants and controls were mated at the same time. The relative fecundity was calculated as the product of the number of litters, the litter size, and the litter interval (in months) per female for a pre-defined period of four months.

### Ovary morphology

Ovaries were removed and fixed in Bouin buffer at 4°C, washed with ethanol, paraffin-embedded and sliced into 7 μm sections. Sections were mounted and deparaffinized, stained with hematoxylin, and coverslipped. The histological analysis of ovary sections was performed to examine the presence of corpora lutea as well as follicles. Follicles with antrum at different stages of folliculogenesis were classified according to their size: 100 to 199 μm (small), 200 to 299 μm (medium), and >300 μm (large).

### Hormone measurements

Females were sacrificed in proestrus for estradiol and progesterone measurement. Blood was collected and circulating levels of estradiol were measured by enzymatic immunoassay using a sensitive estradiol kit (Cayman Chemical Company, Ann Arbor, MI, USA; reference 582251). The intra-assay variation coefficient was 12.9%. Concentrations of plasma progesterone were measured by immunoenzymatic assay with a sensitivity of 0.25 ng/ml. The intra-assay variation coefficient was 15%.

### Mounting behavior (resident-intruder test)

This test was performed as described [22,41]. Briefly, sexually naïve, resident female B6 and cGαo^-/-^ mice (8 to 12 weeks old) were isolated for 10 days. Testing lasted 15 min and began when a sexually inexperienced intruder (either male, female in estrus, or castrated male adult, group-housed) was placed in the home cage of the test mouse (female resident), whose bedding had not been changed for at least four days. Mounting, pelvic thrusts, sniffing, and aggressive behaviors were recorded during the test, included as percentage of animals showing the behavior, latency to first event, cumulative event duration, and number of events. Male mounting behavior was performed similarly using resident male mice (singly housed) instead of female residents and analyzed in response to female intruders in estrus.

### Pregnancy block (Bruce effect)

Adult female mice (50 to 60 days old) were used to assess the pregnancy rates in normal conditions (non-stimulated) and after stimulation with familiar (derived from their original B6 partners) or unfamiliar (BALB/c) male urine after mating. Previously established protocols for mating and stimulation were used [48,53]. Briefly, after one week of isolation in a single cage, adult females in proestrus were mated with an adult, sexually experienced B6 male by introduction of the male in the female’s home-cage for 24 h. After mating, the male subjects were removed from the cage and females were inspected for the presence of plugs. Each female showing a plug was stimulated with 30 μl of familiar or unfamiliar male urine applied onto the oronasal groove every 12 h for three days (five times in total). For unstimulated conditions, females were transferred to a new clean cage after the mating. The first urine application was performed the same day mating was terminated. The subsequent stimulations (four in total) where performed two times a day during the following two days. Ten days after mating, females were sacrificed and subjected to hysterectomy to determine the presence of implanted ova.

### Female sexual receptivity (lordosis)

Adult sexually naive female mice were singly housed for one week and the estrous cycle was determined in order to use only animals in estrus or proestrus prior to the test (see above). Adult males (sexually experienced) were introduced to the female’s home cage and were recorded for 15 min during the dark cycle. The number of mounting behaviors as well as the latency to mount shown by male individuals were scored. The number of lordosis events (in which females show a receptive still posture or arching of the back, allowing or promoting male mounting) by the females was assessed. The lordosis quotient was calculated as the ratio between lordosis events and male mounts, previously described as an index of female reproductive receptivity [2,68].

### Puberty acceleration (Vandenbergh effect)

Young prepubertal female mice (21 days old) were weighed and housed in groups of two or three animals directly after weaning. Females were stimulated twice a day, from postnatal day 23 to 29 (7 days) with male urine, following a previously described protocol [15]. Briefly, 15 μl of urine collected from at least three different adult males was gently applied with a pipette to the oronasal groove of the females. Control females were not stimulated. After the treatment, females were weighed again, euthanized, and the uteri removed. Ovaries and fat tissue were separated and each uterus was then weighed.

### Analysis of female estrous cycle synchronization (Whitten effect)

Adult female mice (50 to 90 days old) were used for a total observation time of 28 days (four weeks). Females were kept in groups of two to three mice per cage to avoid undesired effects of social isolation on hormonal levels and neuroendocrine functions [69]. The estrous cycle of the subjects was monitored daily for two weeks (assessment period) to determine the incidence and frequency of its different phases (proestrus, estrus, metestrus, diestrus). During the stimulation period, a fresh stimulus (piece of filter paper containing 30 μl of male urine) was added to the cage twice a day. At the end of this treatment, the amount of days in estrus and proestrus was calculated as the cumulative receptive days.

### Gender odor preference

Odor preference experiments were conducted with adult female mice (60 to 120 days old) in a custom made 40 × 40 × 40cm plexiglas arena. Each animal was habituated for 30 min to the testing conditions. Mice were then returned to their home cage and re-introduced in the arena 10 min later with filter papers with the odor stimuli (30 μl of male or female urine) located simultaneously in the arena at opposite corners. Sniffing towards the odor source (filter paper) measured as number of sniffing events and total sniffing time was scored in order to assess the relative interest of female mice for one of the two urines.

### Female attraction to familiar versus unfamiliar male scents

This assay evaluated the attraction of a female mouse to male individual volatile scents that were previously experienced during a five-day exposure period, either with or without direct physical access to the odor source [3,4]. This attractive response was based on an olfactory associative learning between volatile and nonvolatile urinary components [3,4,50]. During the exposure step, two urine stimuli obtained from distinct B6 males (50 μl each) were placed on filter paper in the cage housing the female. Whereas one of these filter papers was directly accessible to the female, the second one was placed in a meshed box to prevent direct physical contact with the odor source (Figure 6A). Urine donors were adult B6 males (4 to 5 months old) housed individually, with no kinship relation. Stimuli were delivered randomly two times a day each for 5 min within a total 4 h interval allowing for a 1 h resting phase between stimulations. After this learning period, a recognition step consisting of a two-choice olfactory preference test without physical contact was performed (Figure 6A). Filter papers containing 50 μl of each urine were deposited in meshed plastic boxes to prevent direct physical contact. During a 5 min trial period, stimulus investigation time was scored as the time spent in close contact with the stimulus source (distance of the snout from the box <1 cm) as well as the time spent manipulating, chewing, and biting the meshed box in an attempt to reach the stimulus source. As a critical control to rule out any pre-existing preference prior to the learning phase, we examined whether a given female showed a preference for volatiles in the tested urine sources; only females showing no preference were used for this assay.

### Habituation-dishabituation paradigm

This test was used to measure novel odor investigation, short-term odor learning, and odor discrimination abilities following previously established protocols [41,53,54]. Urine derived from two different B6 male donors was used. Briefly, female mice previously habituated to the testing cage (40 × 16 × 17 cm) for 30 min were familiarized with the urine of the first male in four successive 2 min periods with a 10 min inter-session interval. Mice were then exposed once (2 min) to the second male urine (odor of dishabituation). All stimuli were enclosed in a meshed plastic cassette cage to evaluate volatile odor discrimination only.

### Immunohistochemistry

Mice were anesthetized with a 4:1 cocktail of ketamine and xylazine (Bayer) and perfused transcardially with 0.9% saline solution followed by 0.1 M phosphate buffer (PB) containing 4% paraformaldehyde. The brains were removed, postfixed for 6 h in 4% paraformaldehyde, and incubated overnight in 0.1 M PB containing 30% sucrose. Cryosections (30 μm thick) were mounted on SuperFrost Plus glass slides for immunofluorescence analysis. Tissue sections were washed (10 min) in PBS, incubated in blocking solution containing 0.5% Triton X-100, 4% horse serum, and PBS (1 h, room temperature), and incubated overnight at 4°C in blocking solution containing the first primary antibody. The tissue was then washed in PBS (10 min), followed by incubation in secondary antibody for 1 h at room temperature. Primary antibodies used were anti-Gαo (1:200, rabbit polyclonal; Santa Cruz Biotechnology) and anti-OMP (1:5000, goat polyclonal, Wako Chemicals). Secondary antibodies used were Alexa-Fluor 488 donkey anti-goat and Alexa fluor 555 donkey anti-rabbit (1:1000; Invitrogen).

### Statistics

Independent Student’s t-test was used for measuring the significance of difference between two independent distributions. Paired Student’s t-test was used in Figure 2B-D. Multiple groups were compared using a two-way ANOVA with the Fisher’s LSD as a *post hoc* comparison. Unless otherwise stated, results are presented as means ± standard error of the mean.

## Abbreviations

ANOVA: analysis of variance; AOB: accessory olfactory bulb; B6: C57BL/6 strain mice; CNS: central nervous system; ESP1: exocrine-gland secreting peptide 1; GFP: green fluorescent protein; HMW: high molecular weight; LMW: low molecular weight; LSD: Fisher’s least significant difference; MeA: medial amygdala; MHC: major histocompatibility complex; MOE: main olfactory epithelium; MPOA: medial preoptic area; MUPs: major urinary proteins; NS: non-significant; OMP: olfactory marker protein; PBS: phosphate-buffered saline; PVN: paraventricular nucleus; VNO: vomeronasal organ; VSNs: vomeronasal sensory neurons.

## Competing interests

The authors declare that they have no competing interests.

## Authors’ contributions

LO, AP-G, TL-Z, MK, LB, FZ, and PC designed research; LO, AP-G, MK, EJ and PC performed research; LO, AP-G, MK, EJ, LB, TL-Z, FZ, and PC analyzed data; and LO, TL-Z, LB, FZ, and PC wrote the paper. All authors read and approved the final manuscript.
